# Molecular Profiling of Hard-to-Treat Childhood and Adolescent Cancers

**DOI:** 10.1001/jamanetworkopen.2019.2906

**Published:** 2019-04-26

**Authors:** Fida Khater, Stephanie Vairy, Sylvie Langlois, Sophie Dumoucel, Thomas Sontag, Pascal St-Onge, Henrique Bittencourt, Dorothée Dal Soglio, Josette Champagne, Michel Duval, Jean-Marie Leclerc, Caroline Laverdiere, Thai Hoa Tran, Natalie Patey, Benjamin Ellezam, Sébastien Perreault, Nelson Piché, Yvan Samson, Pierre Teira, Nada Jabado, Bruno Michon, Josée Brossard, Monia Marzouki, Sonia Cellot, Daniel Sinnett

**Affiliations:** 1 Research Centre, Centre Hospitalier Universitaire Sainte-Justine, Montreal, Québec, Canada; 2 Charles-Bruneau Cancer Center, Centre Hospitalier Universitaire Sainte-Justine, Montreal, Québec, Canada; 3 Department of Pathology, Centre Hospitalier Universitaire Sainte-Justine, Montreal, Québec, Canada; 4 Division of Neurology, Centre Hospitalier Universitaire Sainte-Justine, Montreal, Québec, Canada; 5 Department of Surgery, Centre Hospitalier Universitaire Sainte-Justine, Montreal, Québec, Canada; 6 Department of Pediatrics, McGill University, Montreal, Québec, Canada; 7 Division of Hematology-Oncology, Centre Hospitalier Universitaire de Québec, Québec City, Québec, Canada; 8 Division of Pediatric Hematology-Oncology, Centre Hospitalier Universitaire de Sherbrooke, Sherbrooke, Québec, Canada; 9 Department of Pediatrics, Montreal University, Montreal, Québec, Canada

## Abstract

**Importance:**

Little progress in pediatric cancer treatment has been noted in the past decade, urging the development of novel therapeutic strategies for adolescents and children with hard-to-treat cancers. Use of comprehensive molecular profiling in the clinical management of children and adolescents with cancer appears a suitable approach to improve patient care and outcomes, particularly for hard-to-treat cases.

**Objective:**

To assess the feasibility of identifying potentially actionable mutations using next-generation sequencing–based assays in a clinically relevant time frame.

**Design, Setting, and Participants:**

This diagnostic study reports the results of the TRICEPS study, a prospective genome sequencing study conducted in Québec, Canada. Participants, aged 18 years or younger at diagnosis, with refractory or relapsed childhood and adolescent cancers were enrolled from April 2014 through January 2018. Whole-exome sequencing (WES) of matched tumor normal samples and RNA sequencing of tumor were performed to identify single-nucleotide variants, fusion transcripts, differential gene expression, and copy number alterations. Results reviewed by a team of experts were further annotated, synthesized into a report, and subsequently discussed in a multidisciplinary molecular tumor board.

**Main Outcomes and Measures:**

Molecular profiling of pediatric patients with hard-to-treat cancer, identification of actionable and targetable alteration needed for the management of these patients, and proposition of targeted and personalized novel therapeutic strategies.

**Results:**

A total of 84 patients with hard-to-treat cancers were included in the analysis. These patients had a mean (range) age of 10.1 (1-21) years and a similar proportion of male (45 [54%]) and female (39 [46%]). Sixty-two patients (74%) had suitable tissues for multimodal molecular profiling (WES and RNA sequencing). The process from DNA or RNA isolation to genomic sequencing and data analysis steps took a median (range) of 24 (4-41) days. Potentially actionable alterations were identified in 54 of 62 patients (87%). Actions were taken in 22 of 54 patients (41%), and 18 (33%) either were on a second or third line of treatment, were in remission, or had stable disease and thus no actions were taken.

**Conclusions and Relevance:**

Incorporating genomic sequencing into the management of hard-to-treat childhood and adolescent cancers appeared feasible; molecular profiling may enable the identification of potentially actionable alterations with clinical implications for most patients, including targeted therapy and clinically relevant information of diagnostic, prognostic, and monitoring significance.

## Key Points

**Question:**

Can genome sequencing facilitate the molecular profiling of the patient's tumor to identify actionable and targetable alterations?

**Findings:**

In this diagnostic study of 62 consecutive pediatric patients with hard-to-treat cancer who were enrolled in the TRICEPS study, incorporating multimodal genomic sequencing, including RNA sequencing, into the management of refractory or relapsed childhood and adolescent cancers identified potentially actionable alterations in 54 (87%) of patients.

**Meaning:**

Molecular profiling may enable the identification of potentially actionable alterations with clinical implications for most patients tested, including targeted therapy and clinically relevant information of diagnostic, prognostic, and monitoring significance.

## Teaser

This diagnostic study examines next-generation sequencing technologies and their application to the molecular profiling of refractory or relapsed cancers in children and adolescents.

## Introduction

Childhood and adolescent cancers constitute a heterogeneous group of rare diseases. Multicentric clinical trials have led to the continuing refinement of cancer subtype classification and the development of improved risk-adapted treatment strategies, with overall survival rates currently reaching approximately 80%.^1,2^ Despite these advances, cases of refractory and recurrent cancers are associated with a poor prognosis and death. The hard-to-treat cancers remain the leading cause of disease-related mortality among children and adolescents in Western countries.^3,4,5,6^ Little progress has been made to further improve the outcomes of these patients, highlighting the urgent need for new research avenues to tackle this challenge.

The use of comprehensive molecular profiling in the clinical management of children and adolescents with cancer appears a suitable approach to improve patient care and outcomes, particularly for hard-to-treat cases. The advent of next-generation sequencing (NGS) technologies has revolutionized the study of cancers, offering unprecedented opportunities to fully characterize cancer genomes. It has accelerated the search for somatic mutations, which can now be applied to whole genomes and transcriptomes to unravel molecular signatures.^7,8,9,10,11,12^ In-depth molecular profiling of individual tumors has allowed the identification of potentially actionable mutations that could lead to therapeutic interventions and new drug targets.^11,13,14,15,16^ Several initiatives have begun to integrate cancer genomic–based information into the care of patients with childhood cancer. These initiatives have demonstrated the feasibility of such strategies at a single site or across multiple sites.^17,18,19,20,21,22^ They have reported many genomic biomarkers or oncogenic drivers that have been proven useful to tailored patient management.

Working toward this goal, we carried out the TRICEPS study, the personalized targeted therapy in refractory or relapsed cancer in childhood study. The TRICEPS study targeted cases of childhood and adolescent cancer from all 4 pediatric medical oncology centers in the province of Québec, Canada. The primary objective was to assess the feasibility of identifying potentially actionable mutations using NGS-based assays in a clinically relevant time frame. In this current study, we report the results from a cohort of 84 consecutive and clinically well-characterized patients enrolled in the TRICEPS study who underwent extensive molecular profiling. The multimodal genomic and transcriptomic strategies effectively identified various types of genomic alterations, including expressed gene fusions, single-nucleotide variants (SNVs), small insertions/deletions (indels), and copy number alterations (CNAs), that improved the detection of potentially actionable alterations of clinical relevance.

## Methods

### Study Design and Participants

The TRICEPS study, a prospective multimodal genome sequencing study, launched in April 2014 at a single institution, the Centre Hospitalier Universitaire Sainte-Justine in Montreal, Québec, Canada. After a feasibility phase of 2 years (involving patients 1 to 30), the TRICEPS study enrolled patients through January 2018 from all 4 pediatric oncology centers in the province of Québec (Centre Hospitalier Universitaire Sainte-Justine, McGill University Health Centre, Centre Hospitalier Universitaire de Québec–Université Laval, and Centre Hospitalier Universitaire de Sherbrooke). This study was approved by the Research Ethics Board of Centre Hospitalier Universitaire Sainte-Justine. Approved informed consent forms were provided to and completed by all patients. Details of patient enrollment are available in eMethods in the Supplement. This study followed the Transparent Reporting of a Multivariable Prediction Model for Individual Prognosis or Diagnosis (TRIPOD) reporting guideline.

### Molecular Profiling and Data Analysis

#### Whole-Exome Sequencing and RNA Sequencing 

Whole exomes were captured in solution using a kit (SureSelect XT Clinical Research Exome; Agilent) and according to the manufacturer's instructions. Paired-end sequencing (2 × 75 base pairs [bp]) of matched normal and tumor materials was performed on the sequencing system (HiSeq 2500 or HiSeq 4000; Illumina) at the Integrated Centre for Pediatric Clinical Genomics of the Centre Hospitalier Universitaire Sainte-Justine, with an expected mean coverage on targeted region of 300X for tumor and 100X for germline sequences. This coverage allowed the detection of subclonal populations present at 10% or more in tumor material (D.S.; unpublished data; May 2012). To be more time efficient, we performed a first sequence variant analysis on data from a virtual 979 cancer genes panel (eTable 1 in the Supplement). This panel was built from a compilation of genes present in the COSMIC (Catalogue of Somatic Mutations in Cancer) database,^23^ FoundationOne Heme genomic profiling test,^40^ and My Cancer Genome precision cancer medicine tool.^41^ Bioinformatics analysis was performed as described elsewhere.^24^ Details of pipelines used for this analysis are given in eFigure 1 and eMethods in the Supplement.

RNA libraries (TruSeq Stranded Total RNA Library Prep Kit; Illumina) were prepared from cancer cells using a kit (Ribo-Zero Gold kit; Illumina) and according to the manufacturer's protocol. The resulting libraries (stranded and ribosomal RNA depleted) were sequenced (approximately 150 million reads, paired-end 2 × 75 bp) on a sequencing system (either HiSeq 2500 or HiSeq 4000) at the Integrated Centre for Pediatric Clinical Genomics. Details of the bioinformatics analysis performed and the annotation of genomic alterations are available in eMethods in the Supplement.

### Potentially Actionable Alteration Categories and Multidisciplinary Molecular Tumor Board

Further annotation was done using published associations with drug or variant sensitivity profiles (eMethods in the Supplement). Scientific literature was mined to determine if a given alteration was a target of an approved drug or a target of a drug in clinical development, or if it conferred resistance to known treatments. Then, the somatic alterations were ranked with level of evidence (eFigure 2 in the Supplement). This information was integrated to identify potentially actionable alterations, grouped in 1 of 4 categories: targeted therapy, minimal residual disease/biomarker, risk stratification, and diagnostic (eMethods in the Supplement).

The patient’s specific molecular profile was then reviewed by the TRICEPS study’s multidisciplinary molecular tumor board (MMTB), which included experts in pediatric oncology, genomics, bioinformatics, medical genetics, surgery, and pathology. Any treatment decision based on the molecular profiling that outlined potentially actionable alterations or the decision to prescreen patients for ongoing clinical trials was made entirely by the treating team and the patients and their family.

## Results

### Patient Characteristics and Samples

**Figure 1.**
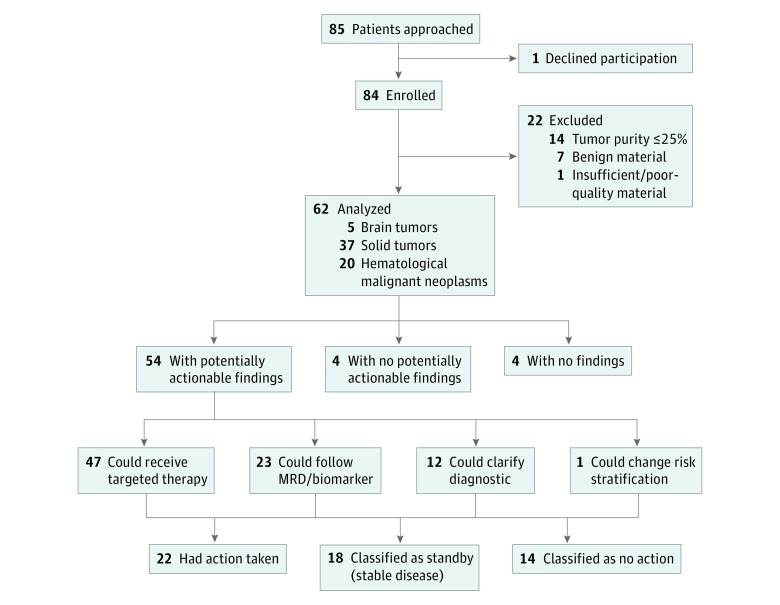
Enrollment Overview and Distribution of Potentially Actionable Alterations MRD indicates minimal residual disease.

A total of 85 consecutive patients with relapsed or refractory or hard-to-treat cancer were eligible, and only 1 patient declined participation. Thus, 84 children and adolescents with various types of cancer diagnosis (Figure 1 and the Table) were enrolled in the TRICEPS study. The sample had a mean (range) age of 10.1 (1-21) years and a similar proportion of male (45 [54%]) and female (39 [46%]).

**Table.** Summary of the Molecular Profiling of 62 Patients

Patient No.	Cancer Type	Tissue Type and Source	Mean Coverage, X		RNA, Million Reads	Potentially Actionable Somatic Alteration	Potentially Actionable Germline Alteration	Category of Potentially Actionable Findings
			WES, Normal	WES, Tumoral				
2	ETP-ALL	Bone marrow	61	76	98	*MLLT10-PICALM* fusion; *KMT2E-ASNS* fusion	NA	Targeted therapy, MRD/biomarker, diagnostic
						del(5q)	NA	Targeted therapy
5	Ewing sarcoma	Left femur biopsy	257	233	NA	ERCC2 F332V	NA	No effect
							SDHD G12S	Genetic counseling
10	Rhabdomyosarcoma	Thigh biopsy	196	194	NA	*PAX7-FOXO1* fusion	NA	MRD/biomarker, diagnostic
						*MDM2* amplification; CDKN2A A102V	NA	Targeted therapy
11	Pilocytic astrocytoma	CNS needle biopsy	191	289	NA	BRAF 507insVLR	NA	Targeted therapy
12	Malignant rhabdoid tumor	Intra-abdominal biopsy	124	455	947	No potentially actionable findings	NA	No effect
13	Medulloblastoma	Cerebellum biopsy	128	383	75	PTCH1 T1195S and *PTCH1* copy loss (LOH), *GLI2* amplification	NA	Targeted therapy
						TP53K132N and *TP53* copy loss (LOH)		No effect
14	AML-M7	Bone marrow	88	507	115	*NUP98-KDM5A* fusion	NA	MRD/biomarker
						*RB1* copy loss	NA	No effect
15	B-ALL	Bone marrow	120	424	362	*CDKN2A/B* homozygous deletion	NA	Targeted therapy
						*DDX5-KLF2* fusion	NA	MRD/biomarker
						TP53 R248Q and *TP53* copy loss (LOH)	NA	No effect
19	B-ALL	Bone marrow (diagnosis)	115	355	172	*PAX5-JAK2* fusion	NA	Targeted therapy, MRD/biomarker, risk stratification
						*CDKN2A* homozygous deletion	NA	Targeted therapy
						PAX5 A322T and *PAX5* copy loss (LOH)	NA	No effect
20	Adrenal gland carcinoma	Adrenal gland biopsy	98	248	178	*AKT1* amplification (4 copies)	NA	Targeted therapy
						*JAK1* copy gain		
						DPYD I543V and *DPYD* copy loss (LOH)		
						TP53 R181H and *TP53* copy loss (LOH)	NA	No effect
21	Osteosarcoma	Left femur biopsy	105	282	168	*MDM2* (6 copies) and *FRS2* (12 copies) amplifications, *AURKA* copy gain	NA	Targeted therapy
						*PMP22-TP53* fusion	NA	MRD/biomarker
22	ETP-ALL	Bone marrow	88	265	131	JAK3 L857P, PHF6 R225X, MED12 S672fs	NA	Targeted therapy
						*KMT2E-ASNS* fusion	NA	Targeted therapy, MRD/biomarker
						*PICALM-MLLT10* fusion	NA	Targeted therapy, MRD/biomarker, diagnostic
24	B-ALL	Bone marrow	107	406	137	BRAF A320V, KRAS G12V, JAK2 R683G	NA	Targeted therapy
25	Hepatoblastoma	Liver biopsy (FFPE sample)	72	230	122	*PRKCA* copy gain, ABL2 and *DDR2* copy gain and high expression, NOTCH1 G1196D, NCSTN A572G, TLR8 N515H	NA	Targeted therapy
26	Osteosarcoma	Right femur biopsy	145	390	66	*MYC* amplification (5 copies)	NA	Targeted therapy
						*TP53* copy loss, *RB1* copy loss	NA	No effect
29	Adrenal gland carcinoma	Adrenal gland biopsy	87	261	164	*PTK2* copy gain, *JAK3* copy gain, AKT2 copy gain, ABL1 A34V, TOP2A A1515S, G1386D (LOH)	NA	Targeted therapy
						NA	TP53p. R337H	Diagnostic, genetic counseling
31	Neuroblastoma	Mediastinum biopsy	100	226	102	Trisomy 7 (*BRAF*), *CHEK1* copy loss, *PRKCA* copy gain, PHOX2B 270-272del frameshift, XPC S346P (LOH), APC D917Y	NA	Targeted therapy
32	AML-M5	Bone marrow	90	252	149	*CBFB-MYH11* and *ICAM2-STX7* fusion	NA	MRD/biomarker
						*TP53* (indel) mutation and copy loss (LOH)	NA	No effect
						Trisomy 8 (*MYC*), NF1 S2309fs and *NF1* copy loss (LOH)	NA	Targeted therapy
33	Paraganglioma	Adrenal gland biopsy	111	308	134	SD*HB* copy loss, DDB2 copy loss, MUTYH Q338H	NA	Targeted therapy
34	Aggressive fibromatosis	Mandible/gums biopsy	60	178	193	CTNNB1 T41A	NA	Targeted therapy
37	B-ALL	Bone marrow	70	206	131	*KMT2A-MLLT1* fusion	NA	MRD/biomarker
39	Ewing sarcoma	Rib needle biopsy	138	372	254	*CDKN2A* copy loss, trisomy 8 (*MYC* and *FGFR1*), BRCA1 mutations (LOH), STAG2 R1012X	NA	Targeted therapy
						TP53 R273H	NA	No effect
						*EWSR1/FLI1* fusion	NA	MRD/biomarker, diagnostic
						NA	SDHD G12S	Genetic counseling
40	Wilms tumor	Kidney needle biopsy	122	381	238	*DDR2* and ABL2 copy gain, DNMT3A P904L	NA	Targeted therapy
47	T-ALL	Bone marrow	90	256	133	*CDKN2A* homozygous deletion, NOTCH1 I1718T and S2467fs, STAT5B N642H, NT5C2 R367Q	NA	Targeted therapy
48	Myeloproliferative neoplasm	Bone marrow	75	179	132	No findings	NA	No effect
49	Rhabdomyosarcoma	Left fornix biopsy	100	252	127	FGFR4 G388R (LOH)	NA	Targeted therapy
						*TP53* copy loss (LOH)	TP53 p.R273C	Diagnostic, genetic counseling
50	Pilocytic astrocytoma	Brain, third ventricle biopsy	73	380	217	FGFR1 656EL, NF1 N1465S, PTPN11 G503A	NA	Targeted therapy
51	Osteosarcoma	Left femur biopsy	134	411	202	*TP53* copy loss (LOH)	TP53 p.G245S (mosaicism)	Genetic counseling
						*CDKN2A* homozygous deletion, *VEGF-A* amplification (>4 copies), *MYC* amplification (>10 copies), JAK2 G996R, CSF1R H362R (LOH), PTK2 exon17:c.1332 + 2T>C (splicing)	NA	Targeted therapy
54	Pilocytic astrocytoma	Optic chiasm–hypothalamus biopsy	142	383	183	*KIAA1549-BRAF* fusion	NA	Targeted therapy, MRD/biomarker
55	Osteosarcoma	Right tibia biopsy	89	285	112	TEK N452D, KIT S590I, *MYC* amplification (10 copies)	NA	Targeted therapy
						*TP53* homozygous del	NA	No effect
57	Neuroblastoma	Abdomen biopsy	125	419	54	*PRKCA* amplification (5 copies), *BRAF* amplification (4 copies), *AKT2* copy gain, HSP90B1 I66T, CSF1R N648S	NA	Targeted therapy
59	Osteosarcoma	Left femur biopsy	135	408	202	*VEGF-A* amplification (4 copies) and highly expressed	NA	Targeted therapy
						*TP53-RAB44* fusion	NA	MRD/biomarker
60	T-Cell lymphoblastic lymphoma	Lymph node	135	404	157	MAP2K2 P128L, NOTCH1 S1674F and Q2503insX, MTOR F1888L, CDKN2A R80X, STAT5B N713insKGKGGG	NA	Targeted therapy
						*KLHL33-TEP1* fusion	NA	MRD/biomarker
61	Osteosarcoma	Left humerus biopsy	128	453	178	*CDKN2A* copy loss, *MYC* copy gain, DDR2 copy gain and highly expressed, MTOR G1954R	NA	Targeted therapy
62	Sinus carcinoma	Sinus biopsy	111	289	163	*PTK2* copy gain, *MYC* copy gain, *ABL2* and DDR2 copy gain, ALK G159fs, NOTCH1 S1674P	NA	Targeted therapy
						*TP53* copy loss	NA	No effect
67	Epithelial tumor (NOS)	Abdomen biopsy	138	430	51	*CREM-FUS* fusion	NA	MRD/biomarker
68	T-ALL	Bone marrow	59	203	65	ABL1 copy gain, DDR2 R742W	NA	Targeted therapy
						*KMT2A-MLLT4* fusion	NA	Targeted therapy, MRD/biomarker
69	Grey zone lymphoma	Lymph node	64	246	70	NA	TP53 p.R213Q	Diagnostic, genetic counseling
70	Leiomyoma	Clavicle biopsy	67	236	55	No potentially actionable findings	NA	No effect
71	Hepatoblastoma	Liver biopsy	62	237	64	No findings	NA	No effect
72	Hepatocarcinoma	Liver biopsy	119	346	120	*DNAJB1-PRKACA* fusion	NA	MRD/biomarker, diagnostic
73	Pleuropulmonary blastoma	Right lung biopsy	113	311	92	*MYC* and *FGFR1* copy gain and highly expressed, *CHEK1* copy loss, CTNNB1 copy gain and very highly expressed, MET Q1276L, DICER1 E1813D	NA	Targeted therapy
						NA	DICER p.Y1225X	Diagnostic, genetic counseling
74	NUT-midline carcinoma	Left fibula needle biopsy	115	354	149	*BRD4-NUTM1* fusion	NA	MRD/biomarker, diagnostic
76	Neuroblastoma	Abdominal needle biopsy	103	318	109	*CDKN2A* homozygous deletion, *MET* amplification (4 copies) and highly expressed, *PHOX2B* copy loss, CHEK1 copy loss, ALK F1245I	NA	Targeted therapy
77	Round cell sarcoma	Soft-tissue left ankle biopsy	109	272	67	*BCOR*exon16 ITD, *DDR2* copy gain	NA	Diagnostic, targeted therapy
78	Rhabdomyosarcoma	Right calf biopsy	113	393	147	*MYCN* amplification (5 copies) and highly expressed, KDM1A G703R (LOH)	NA	Targeted therapy
						*PAX7-FOXO1* fusion	NA	MRD/biomarker, diagnostic
79	Osteosarcoma	Left tibia biopsy (FFPE)	121	367	190	SMO A374E, FBXW7 R465H	NA	Targeted therapy
81	B-ALL	Bone marrow	123	298	143	NRAS G12D, SETD2 E1265fs	NA	Targeted therapy
82	B-ALL	Bone marrow	113	311	215	*IKZF1* deletion, FLT3 Y589D	NA	Targeted therapy
						*ZEB2-CXCR4* fusion and *CXCR4* very highly expressed, SEM*A6A-FEM1C* fusion	NA	MRD/biomarker
86	Metastatic Wilms tumor	Right kidney biopsy	106	287	143	CDC73 M1V, CSF3R G751A, FLT4 A992fs, NTRK1 G607V and H598Y and copy loss (LOH)	NA	Targeted therapy
87	Gastric NET	Celiac lymph node biopsy	99	256	38	*CHEK1* copy loss	NA	No effect
88	Burkitt lymphoma	Abdomen biopsy	115	364	38	B2M M1R, HDAC1 Y303H, *PIK3C2A* splicing mutation, CCND3 R256fs, *PTEN* copy loss	NA	Targeted therapy
						*IGH-MYC* fusion	NA	MRD/biomarker
						TP53 G302fs	NA	No effect
89	B-ALL	Bone marrow	56	134	111	*CDKN2A* homozygous deletion, AURKA copy loss, NRAS G12D	NA	Targeted therapy
91	Teratoma malignant (NOS)	Brain, left ventricle biopsy	103	261	107	No findings	NA	No effect
92	AML	Bone marrow	61	179	134	NRAS G12D	NA	Targeted therapy
						*KMT2A-MLLT3* fusion	NA	MRD/biomarker
93	Ganglioneuroblastoma nodular	Retroperitoneal biopsy	142	340	80	MET K324M, High *ALK* expression	NA	Targeted therapy
94	Lymphoma	Mediastinum biopsy (FFPE)	66	246	NA	*CDKN2A* homozygous deletion,	NA	Targeted therapy
						PTEN L182fs and copy-neutral LOH		
99	AML	Bone marrow	76	248	109	HDAC2 E455fs	NA	Targeted therapy
						*KHDRBS3-ANGPT1* fusion		Targeted therapy, MRD/biomarker
100	Ovarian tumor	Ovary biopsy	82	268	72	CD74 P98S	NA	Targeted therapy
102	Melanotic neuroectodermal tumor of infancy	Periostium skull lesion biopsy (FFPE)	83	170	NA	No potentially actionable findings	NA	No effect
104	Alveolar rhabdomyosarcoma	Right foot biopsy	359	160	77	*PAX3-FOXO1* fusion	NA	MRD/biomarker, diagnostic
						RB1 F650S and copy loss (LOH)	NA	No effect
106	Neuroblastoma	Right adrenal biopsy	131	352	64	*MYCN* amplification (10 copies)	NA	Targeted therapy
							SDHB S163P	Genetic counseling

Abbreviations: AML, acute myeloid leukemia; B-ALL, B-cell acute lymphoblastic leukemia; CNS, central nervous system; ETP-ALL, early T-cell precursor acute lymphoblastic leukemia; FFPE, formalin-fixed, paraffin embedded; LOH, loss of heterozygosity; MRD, minimal residual disease; NA, not applicable; NET, neuroendocrine tumors; NOS, not otherwise specified; NUT, nuclear protein of the testis; T-ALL, T-cell acute lymphoblastic leukemia; VEGF-A, vascular endothelial growth factor A; WES, whole-exome sequencing.

Tissues were suitable for molecular profiling in 62 of 84 eligible patients (74%). The mutations in 62 patients were drug-targetable alterations (47 [76%]), alterations that modify diagnosis or risk stratification (13 [21%]), or alterations with a potential for disease monitoring (23 [37%]). For patient 19 (pre–B-cell acute lymphoblastic leukemia), we sequenced the primary leukemia sample because of low blast count (≤25%) in the relapsed sample; then, we confirmed the results in the relapsed material. Five solid tumor samples (patients 5, 10, 11, 94, and 102) were not subjected to transcriptomic analysis because of poor RNA quality (RNA integrity number values <5) or not enough RNA. The remaining 22 patients (26%) were considered as screening failure, owing to benign or necrotic biopsies (n = 7), low tumor content (≤25% tumor purity; n = 14), or insufficient material (n = 1), resulting in suboptimal DNA/RNA quantity or quality suitable for NGS (Figure 1).

### Overall Genomic Alterations Detected by Whole-Exome Sequencing and RNA Sequencing

For whole-exome sequencing (WES), the median (range) coverage depth was 294X (76X-506X) for the tumoral exomes and 106X (54X-256X) for the normal exomes (the summary of sequencing depth is presented in the Table). The analysis of a virtual 979 cancer gene panel (eTable 1 in the Supplement) from the WES data was prioritized to identify somatic genetic changes in tumors (the molecular profiling findings are summarized in Figure 1 and the Table).

**Figure 2.**
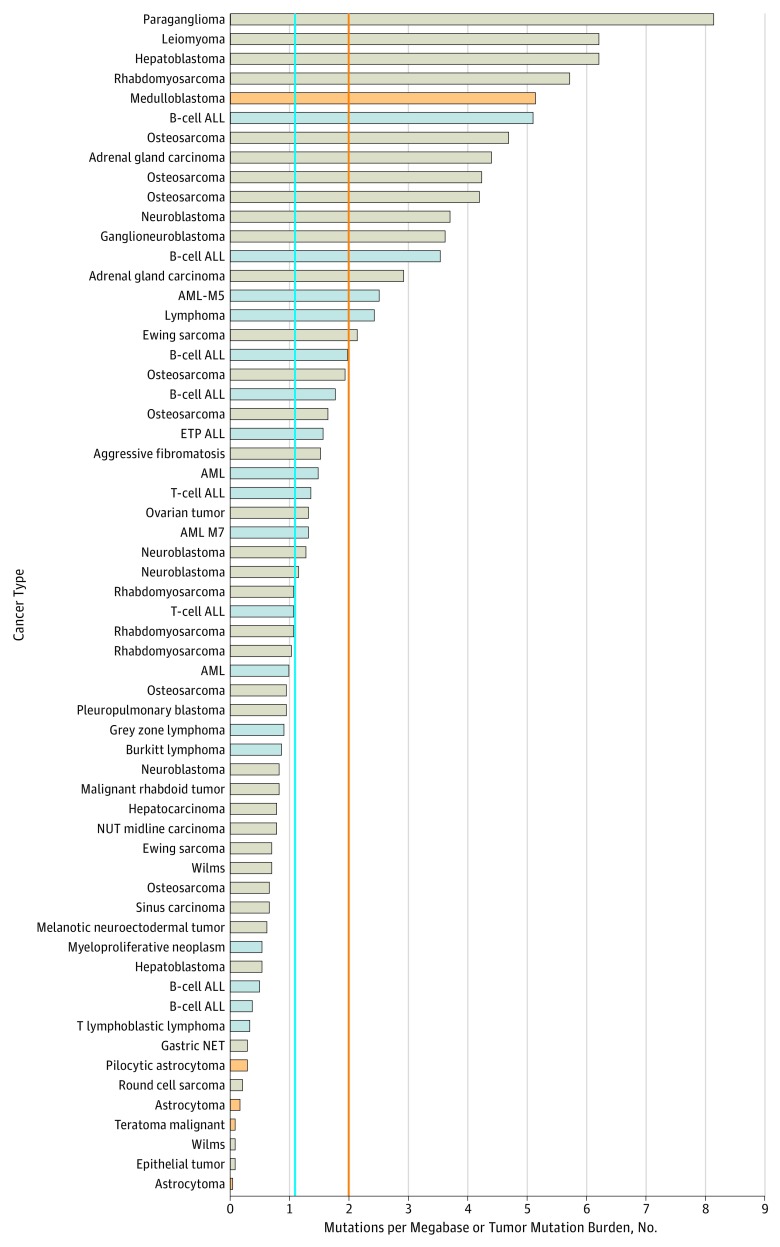
Tumor Mutation Burden for 62 Patients Distribution of the somatic tumor mutation burden is defined as the number of nonsynonymous coding mutations per megabase. Each bar indicates the mutation number in each sample. The blue line indicates the median of the TMB in the cohort (1.09), and the orange line indicates pediatric high threshold, as determined by Gröbner et al.^25^ Solid tumors are labeled in tan, brain tumors in orange, and hematological malignant neoplasms in blue. ALL indicates acute lymphoblastic leukemia; AML, acute myeloid leukemia; ETP, early T-cell precursor; NET, neuroendocrine tumors; NUT, nuclear protein of the testis.

The comprehensive genomic analysis detected at least 1 potentially actionable alteration in 54 of 62 patients (87%) (Figure 1). Among these alterations (n = 191), missense mutations were the most frequent (73 [38%]), whereas mutations that resulted in indels, prematurely truncated proteins, or splicing site changes made up most (15 [8%]) of the remaining alterations (Table). All potentially actionable alterations were verified through orthogonal methods (MiSeq sequencing, quantitative polymerase chain reaction [PCR], or reverse-transcriptase PCR). The tumor mutation burden (TMB), a measurement of the overall number of mutations carried by tumor cells, assessed from WES data (Figure 2) ranged from 1 or lower to 8 with a mean (SD) of 1.87 (1.87) and a median of 1.09. Approximately 10% of patients (6 of 59) had a TMB higher than 5, which may gauge a response to immunotherapy agents.^26^ Copy number alterations were found in 12 genomic regions containing genes present in the virtual 979 cancer gene panel (eTable 1 in the Supplement). Quantitative PCR validation indicated that detection of CNAs was highly concordant with results obtained using standard techniques, including fluorescence in situ hybridization. These results demonstrate the power of the WES-based assay to detect cancer-associated CNAs. Using the germline WES data, we identified 8 (13%) of 62 patients carrying 1 germline pathogenic variant in the virtual cancer predisposition gene (eTable 2 in the Supplement).

For RNA sequencing (or whole-transcriptome sequencing), the mean (range) coverage depth was 140 million (38.4 million-362 million) reads. Using this data set, we detected at least 1 expressed gene fusion in 23 of the 57 patients (40%) tested, mostly in leukemias (12 of 18) and sarcomas (6 of 13) (Table). RNA sequencing was also used to validate the presence of 93% of the SNVs (n = 88) and all estimated splicing mutations (n = 2) found within WES data. Thus, RNA sequencing can be used as orthogonal validation of expressed WES data.

### Clinical Relevance of the Potential Actionable Alterations

The median (range) time frame from patient enrollment to MMTB meeting was 8.76 (4.6-17.1) weeks, including 24 (4-41) calendar days from NGS to data annotation. At least 1 potentially actionable alteration was found in 54 of 62 patients (87%) (Figure 1 and Table). These alterations might either have changed the initial diagnosis (for 12 of 54 patients [22%]) or refined the risk stratification (for 1 of 54 patients [2%]) (Figure 1). In 23 patients (43%), we found at least 1 expressed gene fusion that could be used to detect and follow minimal residual disease (MRD). In 47 of 54 patients (87%), we found at least 1 mutation (or associated pathways) that could be targeted by a US Food and Drug Administration–approved drug or a drug in clinical trial. Nine of these 47 patients (19%) received a targeted therapy according to the molecular profile. For 2 additional patients, the targeted therapy based on the alterations detected was already part of the treatment received (Box). In addition, 18 of the 54 patients (33%) were on a second or third line of treatment, were in remission, or had stable disease at the moment of the report delivery. They were classified as standby as no action had been taken yet (Figure 1). Furthermore, 5 of the 62 patients (8%) analyzed died before the end of the process.

Box. **Summary of Actions Taken Based on Detected Potentially Actionable Alterations by Patient Identification Number and Tumor Type**

**2 ETP-ALL:** Patient has been reclassified as an ETP-ALL. RT-PCR assay was designed for *MLLT10-PICALM* fusion MRD follow-up.
**14 AML-M7:** RT-PCR assay was designed for *NUP98-KDM5A* fusion MRD follow-up.
**15 B-ALL:** RT-PCR assay was designed for *DDX5-KLF2* fusion MRD follow-up. Proposed immunotherapy according to *TP53* mutation and LOH.
**19 B-ALL:** RT-PCR assay was designed for *PAX5-JAK2* fusion MRD follow-up. The patient was reclassified as having a Ph-like ALL (very high risk). Ruxolitinib phosphate given to avoid GVHD.
**20 Adrenal gland carcinoma:** Chromosomal instability in the tumor and *TP53* R181H and LOH; referring physician suggested complete surgery.
**22 ETP-ALL:** The patient was reclassified as an ETP-ALL, and the treatment plan has changed consequently. *PICALM-MLLT10* was used for MRD follow-up.
**24 B-ALL:** Ruxolitinib has been used to avoid GVHD for 1 month according to *JAK2* mutation, and then stopped because of encephalopathy. Sirolimus was also used to avoid GVHD.
**25 Hepatoblastoma:** Dasatinib was given as a single agent based on *DDR2* gain and high expression.
**32 AML-M5:** Sirolimus was used to avoid GVHD according to *NF1* mutation and LOH.
**34 Aggressive fibromatosis:** Patient was already receiving celecoxib.
**39 Ewing Sarcoma:** The patient was referred to the medical genetics division for familial investigation according to *TP53* mutation.
**47 T-ALL:** Nelarabine and dasatinib have been added to treatment for the third induction according to *NT5C2* and *STAT5B* mutations.
**49 Rhabdomyosarcoma:** The patient received pazopanib hydrochloride in monotherapy according to the *FGFR* mutation.
**50 Pilocytic astrocytoma:** The patient is receiving trametinib dimethyl sulfoxide based on *NF1* mutation and shows partial response after 8 months.
**54 Pilocytic astrocytoma:** The patient was already on a clinical trial for MEK inhibitor according to the *KIAA1549-BRAF* fusion detected in clinic.
**57 Neuroblastoma:** Dabrafenib mesylate was proposed off study to the family. Private insurance would have covered the cost, but parents decided not to go with the treatment.
**59 Osteosarcoma:** Pazopanib was started as monotherapy according to *VEGF* expression but was stopped for adverse effects.
**69 Grey zone lymphoma:** The patient was referred to the medical genetics division for familial investigation according to *TP53* mutation.
**73 Pleuropulmonary blastoma:** Celecoxib was added to therapy according to *CTNNB1* very high expression. Sorafenib was started for progression according to *FGFR1* high expression.
**74 NUT-midline carcinoma:** Diagnosis was changed to NUT-midline carcinoma following *BRD4-NUTM1* fusion identification.
**77 Round cell sarcoma:** Confirmation of the diagnosis of a rare subtype of round cell sarcoma of infancy that is not responsive to chemotherapy. Complete resection was achieved.
**82 B-ALL:** *CXCR4* high expression used for MRD.

Abbreviations: AML, acute myeloid leukemia; B-ALL, B-cell acute lymphoblastic leukemia; ETP-ALL, early T-cell precursor acute lymphoblastic leukemia; GVHD, graft-vs-host disease; LOH, loss of heterozygosity; MRD, minimal residual disease; NUT, nuclear protein of the testis; RT-PCR, reverse transcriptase polymerase chain reaction; T-ALL, T-cell acute lymphoblastic leukemia; VEGF, vascular endothelial growth factor.

The alterations in the targeted therapy category were mostly identified by the WES, whereas the MRD/biomarker and risk stratification categories were identified by RNA sequencing, illustrating the power of a multimodal strategy. Of the 12 alterations used for diagnostic, 8 (67%) were detected within the molecular clinical laboratory analysis, particularly fusion products in solid tumor and germline mutations, whereas most of the druggable alterations were detected only by molecular profiling.

**Figure 3.**
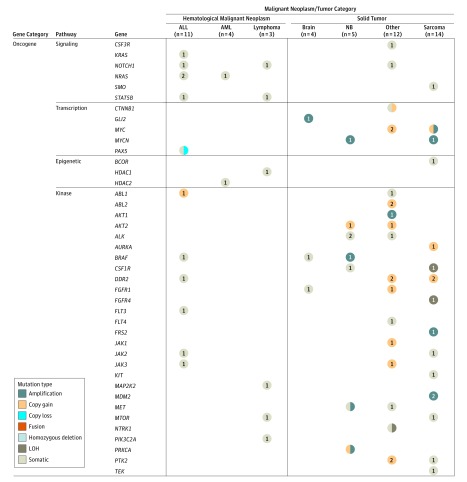
Summary of the Molecular Profiling of Patients in Oncogene Gene Category Data were derived from all 62 patients with completed whole-exome sequencing as well as RNA sequencing of tumors and whole-exome sequencing of germline DNA. The presence of specific mutations, insertion/deletions (indels), amplification/deletions, and genes fusions are indicated by colored circles for hematological malignant neoplasms and solid tumors. Only sequencing findings with biological significance are included. Somatic type included somatic single-nucleotide variants or indels. Sarcoma included rhabdomyosarcoma, Ewing sarcoma, osteosarcoma, round cell sarcoma. Brain type included pilocytic astrocytoma medulloblastoma. Other types included malignant rhaboid tumor, adrenal gland carcinoma, hepatoblastoma, paraganglioma, Wilms tumor, sinus carcinoma, hepatocarcinoma, pleuropulmonary blastoma, NUT midline carcinoma, epithelial tumor, and gastric NET. ALL indicates acute lymphoblastic leukemia; AML, acute myeloid leukemia; LOH, loss of heterozygosity; NB, neuroblastoma; NET, neuroendocrine tumors; NUT, nuclear protein of the testis.

**Figure 4.**
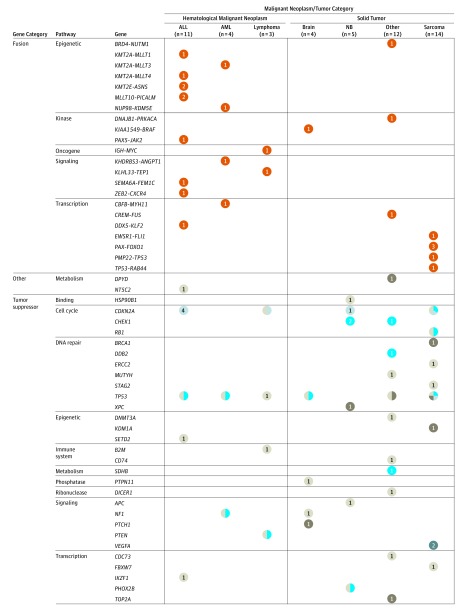
Summary of the Molecular Profiling of Patients in Fusion, Other, and Tumor Suppression Gene Category Data were derived from all 62 patients with completed whole-exome sequencing as well as RNA sequencing of tumors and whole-exome sequencing of germline DNA. The presence of specific mutations, insertion/deletions (indels), amplification/deletions, and genes fusions are indicated by colored circles for hematological malignant neoplasms and solid tumors. Only sequencing findings with biological significance are included. See the caption to Figure 3 for the types included in each neoplasm/tumor category and for the color key.

The alterations identified in this study were not exclusive, as illustrated by patients carrying several mutations in their cancer (Table). Some genes or pathways were frequently altered (Figure 3 and Figure 4; eFigure 3 in the Supplement). For example, tumor suppressors such as *TP53* (7157) and *CDKN2A* (1029) were altered in 19 (31%) of 62 patients analyzed. Oncogenic kinases were altered in 23 of 62 patients (37%). Two sarcomas (patients 10 and 21) had an *MDM2* (4193) amplification (≥6-fold) that could be targeted by *MDM2* inhibitors.^27,28^ The *JAK1/2* (3716) signaling pathway was altered in 6 patients (patients 19, 20, 22, 24, 29, and 51) who could have been treated with *JAK1/2* inhibitors. Some of the fusion gene products putatively participated in key pathways, such as MAPK pathway (patients 2, 22, 54, and 68), *TP53* (patients 21 and 59), and AKT/mTor pathway (patients 29 and 106).

### Clinical Action Taken and Outcomes

The clinical implications of these potentially actionable alterations for treatment decisions and/or outcomes were discussed and recorded following the MMTB review and recommendations. Actions were taken for 22 (41%) of 54 patients in all 4 categories (Figure 1). A summary of the action taken is given in the Box, and representative examples are discussed here.

Disease-specific alterations were useful for targeted therapies as for patient 50,^29^ who had a pilocytic astrocytoma diagnosis and who received a targeted treatment with a MEK inhibitor (trametinib dimethyl sulfoxide) according to the molecular profiling analysis, which uncovered a mutation in *NF1* (N1465S; 4763), a negative regulator of the Ras signal transduction pathway. Patient 50 remained clinically stable, and the last radiologic evaluation after 8 months of treatment showed a decrease in size and enhancing of the primary mass.

Patient 19 underwent a risk stratification change based on the *PAX5* (5079)-*JAK2* (3717) fusion identified by transcriptome analysis. This rearrangement is reported in Ph-like acute lymphoblastic leukemia subtype, a particularly aggressive subtype.^30^ The molecular profiling has changed the diagnosis for patient 73, who initially received a Ewing-like sarcoma diagnosis on the basis of histologic appearance and immunohistochemistry, but the *EWSR1* (2130) and *FUS* (2521)–derived fusions were not detected by fluorescence in situ hybridization analysis. Transcriptome analysis revealed a *BRD4* (23476)-*NUTM1* (256646) fusion, which is exclusively reported in the very aggressive NUT (nuclear protein of the testis)–midline carcinoma, and therefore the diagnosis was changed consequently.

Several alterations were used for MRD monitoring. We identified the expression of at least 1 fusion gene in 12 patients with leukemia. In 9 cases, the expressed gene fusions were not detected in the clinical setting on the basis of targeted reverse transcriptase PCR, standard fluorescence in situ hybridization, or cytogenetic analysis. We developed reverse transcriptase PCR–based assays for 4 of these fusion genes (*PICALM* [8301]*-MLLT10* [8028], *NUP98* [4928]-*KDM5A* [5927], *PAX5-JAK2*, *DDX5* [1655]-*KLF2* [10365]) to allow MRD follow up for these patients. One of the patients died before discussing the results. The TRICEPS study analysis revealed, among others, the presence of the *KMT2E* (55904)-*ASNS* (440) fusion in conjunction with a cryptic t(10;11)(p13;q21) that was missed by conventional cytogenetics within the tumoral material in both patient 22 and patient 2.^14,16^ These findings would have changed the stratification from the diagnosis, and patients would have been treated on a higher-risk arm (very high risk).

## Discussion

The TRICEPS study started as a feasibility study at a single institution to build a precision medicine program that integrates genomic data into clinical decision making. It was designed as a multimodal assay (WES and RNA sequencing) to reliably identify clinically relevant information derived from genomic profiling (SNV, indels, CNAs, gene fusions, and TMB) to guide personalized patient management. It was a comprehensive molecular profiling program, compared with similar ongoing studies (eTable 3 in the Supplement). Patients enrolled in the TRICEPS study had advanced or metastatic cancer that was refractory to standard therapy, had relapsed after standard therapy, or had cancer for which no standard therapy was available. All eligible patients were recruited without regard to the probability of success. This cohort of consecutive patients provided a realistic insight into the distribution of patients with hard-to-treat cancers who could gain an advantage from molecular profiling in a clinical setting.

The present study showed that the molecular profiling (RNA libraries, sequencing, and bioinformatics analysis) of 62 of the 84 enrolled patients could be completed in a clinically reasonable median (range) time frame of 24 (4-41) days. This time frame is comparable to the median turnaround times, ranging from 28 to 54 days, in other studies.^17,22^ Differences in the techniques used for the molecular profiling (WES, gene panel, RNA sequencing) explain most of the observed discrepancy between studies. Screening failures occurred in 22 patients because the tumor content was less than 25% or sufficient material was lacking. In comparison, other studies required a tumor content of at least 40% based on histologic assessment.^22^ By integrating both WES and RNA sequencing, we identified potentially actionable alterations in 87% of the patients analyzed. Most of these mutations had not been detected by molecular testing as part of routine clinical care. These mutations in 62 patients were drug-targetable alterations, alterations that modify diagnosis or risk stratification, or alterations with a potential for disease monitoring; these findings are comparable with those in similar studies (eTable 3 in the Supplement).

In addition to detecting SNVs and indels, WES enabled the identification of additional genomic events, including CNAs and TMB status, which may provide patients with treatment options that would have otherwise been missed. The TMB is a potential biomarker to evaluate response to immunotherapy.^31,32^ As expected, we observed that the overall mutational burden in children and adolescents with cancer was lower than in adults with cancers.^33,34^ The reported median TMB at age 10 years is 1.67 mutations/Mb (megabase),^34^ but some tumors had a mutational burden above the mean (2-8 mutations/Mb) and could be referred to as pediatric highly mutated.^25^ Whether these highly mutated pediatric tumors are candidates for immunotherapy remains to be investigated.

The inclusion of RNA sequencing provided valuable insights, particularly in leukemias and sarcomas, by detecting expressed fusion genes leading to new diagnoses, novel gene fusions, and new treatment options. Of the 57 cases in which RNA sequencing was performed, expressed fusions were detected in 40%, which led to the identification of 25 potentially actionable alterations. In this regard, patient tumors expressing oncogenic fusion proteins tended to be particularly sensitive to targeted inhibition of the fusion protein. One of the best examples is the treatment of leukemia with agents that affect Bcr-Abl kinase activity.^35,36^ These RNA sequencing discoveries alone accounted for approximately 18% of the potentially actionable findings in this study, illustrating the added value of transcriptome analysis. The use of RNA sequencing to identify actionable expressed gene fusions has also been demonstrated in the INFORM^22^ and the PEDS-MIONCOSEQ^17^ studies.

We identified recurrent mutations in genes or related pathways, including tyrosine kinases, *JAK-STAT* gene, AKT/mTor pathway, MAPK pathway, and tumor suppressors (*TP53* and *CDKN2A*), that could be targeted by approved drugs. The MMTB identified 47 patients that could receive targeted therapy, 9 (19%) of whom were treated with the proposed alternative therapy. This percentage is similar to those reported by other studies, ranging from 3% to 19%.^17,19,22^ The main barriers to the administration of targeted therapy in these patients included results that were available too late in the clinical course, limited drug access (regulatory and cost issues), and lack of an available clinical trial. In addition, we identified alterations, including novel alterations of previously unknown significance that have now been further characterized.^14,16^

The yield of detection of potentially actionable alterations achieved in the TRICEPS study is in the upper range (87%) as compared with similar studies (eTable 3 in the Supplement). The integration of WES-based methods to detect CNAs and RNA sequencing to identify fusion genes has increased the yield of potentially actionable alteration detection. The number of potentially actionable alterations identified may possibly have been overestimated, or other studies might have missed actionable mutations. This discrepancy could be partly explained by the lack of a standard definition for an actionable alteration and the level of evidence needed to support it. For instance, several studies considered only druggable genomic alterations, whereas other studies, including the TRICEPS study, recognized that nondruggable alterations might also be actionable or clinically relevant (eg, impact diagnosis, prognosis, or risk stratification). Other reasons for this discrepancy may include different cohort sizes, variable inclusion criteria, and investigation of specific cancer subgroups. In addition, the molecular profiling design, bioinformatics pipelines, and data analyses varied between studies. For instance, some precision medicine trials (eTable 3 in the Supplement) were focused on specific cancer subtypes (eg, non–central nervous system solid tumors) and had limited genomic investigations (eg, gene panel).

Up to 10% of pediatric patients with cancer are estimated to carry an underlying hereditary cancer predisposition gene,^37^ making the discovery of clinically relevant germline variants^38^ inevitable during NGS analysis using germline SNVs to distinguish cancer-specific somatic mutations from constitutional variants. In the TRICEPS study, we detected pathogenic or likely pathogenic germline variants in 8 patients (13%), and this information was reported to the referring clinician. Patients and their families were then offered a referral to the medical genetics division for genetic counseling. In the future, we plan to use the WES data from the normal genome to assess interindividual variability in drug-related genes involved in absorption, distribution, metabolism, and excretion, which will allow us to estimate an individual’s drug response and toxicological profile.

A key to the success of the TRICEPS study was the role played by the MMTB, which discussed, critiqued, and deliberated on molecular profiles and their actionable potentials. The NGS data were synthesized into a report that focused on putative actionable alterations, related pathways, and therapeutic alternatives. This report provided information to be used at the treating physician’s discretion. The molecular profiling data were not meant to be prescriptive, but rather they were intended to provide novel information to guide the management of individual patients with cancer. The MMTB not only discussed the actionable potential of the molecular findings but also shared clinical, regulatory, and ethical issues associated with the findings. These MMTB meetings also served as a platform to train the next generation of scientists, clinicians, and other health professionals in the field of genomics to better understand the application of NGS data in a tailored-treatment strategy. Because of major improvements in caring for children and adolescents with hard-to-treat cancer, studies such as the TRICEPS study will likely become more frequent. Thus, exploring the ethical issues associated with these studies is important. More than 90% of parents reported that taking part in the TRICEPS study was advantageous for several reasons, but mainly it gave their children “all their chances” and an opportunity to give back (ie, improve care for future patients).^39^

### Limitations

This study is limited by the small numbers included. In addition, the heterogeneous nature of the patients made drawing general statements difficult. Because the purpose of this study was not to follow up with the patients, assessing the long-term implications of the actions taken was not possible.

## Conclusions

The present study appeared to demonstrate the feasibility of a comprehensive and real-time molecular profiling to identify actionable alterations in nonselected hard-to-treat childhood and adolescent cancers. Despite the challenges associated with translating genomic cancer landscape discoveries into a clinical setting, the TRICEPS study has shown its value to therapeutic management, including treatment options and diagnoses that change patient outcome. By generating clinically actionable findings, the TRICEPS study is establishing processes for incorporating NGS into routine cancer care. The development of standardized definitions for clinical categorization of somatic mutations will be critical to conducting comparative analyses between different genomic testing platforms and patient populations.

## Notes

Supplement. **eMethods.** Study Details **eFigure 1.** Schematic Illustration of the Bioinformatics Pipelines Used for Genomic-based Molecular Profiling **eFigure 2.** Ranking of TRICEPS Actionable Alterations **eFigure 3.** Distribution of Molecular Alterations Considered as “Potentially Actionable” in Targeted Pathways **eTable 1.** Virtual 979 Cancer Gene Panel Used in TRICEPS to Detect Somatic Mutations **eTable 2.** Virtual 112 Cancer Predisposition Gene Panel Used in TRICEPS to Detect Germline Variants **eTable 3.** Summary of the Genomic Sequencing Initiatives in Pediatric Oncology **eReferences**
