# Supplementary material for: Molecular Profiling of Hard-to-Treat Childhood and Adolescent Cancers
Source: JAMA Netw Open. 2019 Apr 26;2(4):e192906. doi: 10.1001/jamanetworkopen.2019.2906 (PMC6487576; doi:10.1001/jamanetworkopen.2019.2906)
Supplement: Supplement. — eMethods. Study Details eFigure 1. Schematic Illustration of the Bioinformatics Pipelines Used for Genomic-based Molecular Profiling eFigure 2. Ranking of TRICEPS Actionable Alterations eFigure 3. Distribution of Molecular Alterations Considered as “Potentially Actionable” in Targeted Pathways eTable 1. Virtual 979 Cancer Gene Panel Used in TRICEPS to Detect Somatic Mutations eTable 2. Virtual 112 Cancer Predisposition Gene Panel Used in TRICEPS to Detect Germline Variants eTable 3. Summary of the Genomic Sequencing Initiatives in Pediatric Oncology eReferences [file jamanetwopen-2-e192906-s001.pdf]

Supplementary Online Content

Khater F, Vairy S, Langlois S, et al. Molecular profiling of hard-to-treat childhood and adolescent cancers. *JAMA Netw Open.* 2019;2(4): e192906. doi:10.1001/jamanetworkopen.2019.2906

eMethods. Study Descriptions
eFigure 1. Schematic Illustration of the Bioinformatics Pipelines Used for Genomic-based Molecular Profiling
eFigure 2. Ranking of TRICEPS Actionable Alterations
eFigure 3. Distribution of Molecular Alterations Considered as "Potentially Actionable" in Targeted Pathways
eTable 1. Virtual 979 Cancer Gene Panel Used in TRICEPS to Detect Somatic Mutations
eTable 2. Virtual 112 Cancer Predisposition Gene Panel Used in TRICEPS to Detect Germline Variants
eTable 3. Summary of the Genomic Sequencing Initiatives in Pediatric Oncology
eReferences

This supplementary material has been provided by the authors to give readers additional information about their work.

eMethods. Study Descriptions

Study design and participants

Children and adolescents with refractory or recurrent cancer and who were less than 18 years at initial diagnosis were considered eligible for the study. Exclusion criteria included estimated life expectancy of less than 3 months, as assessed by the treating oncologist, and insufficient or low-quality tumor samples. From April 2014 to February 2018, 85 consecutive patients with either a solid, brain or hematological neoplasms with poor prognosis were eligible. The institutional review board approved the research protocol and written informed consent was obtained from all participants and their parents or legal guardians. It is important to note that such studies, in patients with a very reserved prognosis, raise many ethical issues, including the risk of giving "false hope", the risk related to the biospy that would not result in any change in treatment, delaying the decision of comfort care, spending more time in the hospital, and the risk of incidental findings. A companion ethical study was designed and conducted to explore these issues in depth (Janvier et al, in preparation). Clinical and demographic data of patients, including age, sex, disease status at enrolment, were collected at time of enrolment. Biological material as well as patient data were stored in our institutional biobank and database.

Sample and clinical data collection

Following consent, normal and tumor patient samples were obtained. For solid and brain tumors, 3 ml of peripheral blood (Vacutainer blood EDTA tubes) or Saliva (ORAGENE OG-SOO kits) was collected as normal material. Since the protocol did not mandate biopsy for research purposes only, tumoral tissue was obtained following biopsy or resection following routine clinical procedures. When fresh or frozen tissue was not available, Formalin-Fixed

©2019 Khater F et al. *JAMA Network Open.*

Paraffin-Embedded (FFPE) blocks were used instead. Clinical pathologists reviewed all tumor specimens to determine tumor cell content and overall quality of the specimen. Genomic profiling required at least 5 mg of tumor tissue and >25% tumor content. Decalcified specimens were considered inadequate for tumor profiling. For hematologic malignancies, saliva was collected using the ORAGENE OG-5OO or SC-2 kits as normal material. Cancer cells were obtained from either bone marrow, pleural fluid or peripheral blood (Vacutainer blood EDTA tubes). Leukemia samples with more than 25% blasts and at least 100 000 cells were considered suitable for molecular profiling. Genomic DNA and total RNA were extracted from the patient's tumor and normal cells using mini or micro AllPrep DNA/RNA kits from Qiagen, or ORAGENE OG-250/500 or CS-2 protocols for saliva specimens.

Molecular profiling and data analysis

Whole exome sequencing (WES)

Bioinformatic analysis was performed as described elsewhere [1]. Details of pipelines used for bioinformatics analysis are given in Figure S1 (supplementary materials). Briefly, the resulting exome reads were aligned to the hg19 (GRch37) reference genome using BWA (version 0.7.7) [2]. Picard (http://picard.sourcefourge.net) was used to remove duplicate mappings, calculate metrics and manipulate SAM/BAM files. Base quality score recalibration and local realignment of reads around small insertions/deletions (InDels) were performed using the Genome Analysis ToolKit (GATK Version 3.3) [3]. SNVs and InDels were called using Varscan2 [4] and MuTect (https://www.nature.com/articles/nbt.2514). The sequencing information from the corresponding germline genome was used to confirm the somatic status of the mutations. The tumor specific SNVs and indels were considered validated if detected by both WES and transcriptome analysis, otherwise they were confirmed by targeted sequencing (> 1000x coverage) on a MiSeq Illumina system (at the McGill University and Genome Quebec

Innovation Center). CNAs were detected, by selecting off-target reads to simulate a low coverage WGS (Sinnett, unpublished results) and then using the R package QDNAseq [5]. Validation of CNAs was done by qPCR. Tumor mutation burden (TMB), defined as the rate of SNVs per megabase, was determined for all tumors.

Whole Transcriptome sequencing (RNA-seq)

Alignment to the hg19 (GRCh37) genome reference was performed using STAR aligner [6]. Gene expression was measured with the cufflinks software using the Ensembl version 75 gene coordinates. Single nucleotide variants (SNVs) and small insertions/deletions (InDels) were identified using the HaplotypeCaller software included in the Genome Analysis Toolkit (GATK) developed at the broad institute. Fusion genes, translocations and chimeric transcripts were identified with FusionCatcher [7]. STAR Fusion (doi.org/10.1101/120295) was used to rank putative reciprocal breakpoints from the STAR output. UCSC genome browser [8] and Blat [9] were used for visual inspection and evaluation of chimeric transcripts. The identified expressed fusions were validated by reverse transcription polymerase chain reaction (RT-PCR).

Annotation of genomic alterations

SNVs and small indels were called from Bam files using a combination of callers, including Mutect and Varscan comparing the tumor genome with the normal counterpart. In addition, the resulting somatic mutations were screened against 1000 Genomes [10], NHLBI ESP data (evs.gs.washington.edu/EVS/), and our in-house database of normal exomes to filter out variants with minor allele frequency >0.01. ANNOVAR [11] and Oncotator [12] were used to annotate somatic splice site variants, non-synonymous SNVs and frameshift indels. To enrich for putative pathogenic driver genes, the predicted functional impact of non-synonymous variants and small indels were assessed using Sift (version 1.03) [13], Polyphen2 (version 2.2.2;

©2019 Khater F et al. *JAMA Network Open.*

[14], and CADD [15] as previously described [16]. Any variants classified as benign or likely benign were excluded. The joint variant calling approach provided a sensitive determination of somatic alterations, assessed the extent of normal cell contamination, and provided the basis for inferring a ploidy model[17] for the tumor. The driver potential of the genes/variants was further assessed based on their occurrence in public cancer databases such as COSMIC. Somatic gene mutations overlapping with the NCI's TARGET (http://target.cancer.gov) and the Pediatric Cancer Genome Project (PCGP) (http://www.pediatriccancergenomeproject.org) datasets were also prioritized. The somatic mutations were catalogued into the following fashion: (1) Variants in a gene of the virtual Cancer gene panel (979 genes, Table S1); (2) Non-synonymous, Indel, stop gain/loss or splicing variants; (3) Variants with damaging prediction by SIFT and Polyphen2 databases. (4) Expressed splicing isoforms and gene fusion.

Analysis of the germline variant content was performed on data from a virtual cancer predisposition gene panel (112 genes, Table S2) based on the literature review [18], public databases including ClinVar, the Human Genome Mutation Database and Variant specific databases. Only known pathogenic variants based on ClinVar were considered for disclosure if clinically indicated.

Categorization into potentially actionable alterations.

Drug Gene interaction database (http://dgidb.genome.wustl.edu/), DrugBank (http://www.drugbank.ca/), clinical trials (ClinicalTrial.gov) as well as the Pharmacogenomics Knowledgebase (https://www.pharmgkb.org/), FDA (http://www.fda.gov/drugs/), and Health Canada (http://www.hc-sc.gc.ca/) were used for further annotation.

The 4 categories defined to classify the putative actionable alterations were: (1) Eligibility for targeted therapy: genes or pathways that could be targeted by a drug (drug repositioning); (2)

Minimal residual disease (MRD)/Biomarker: alterations (e.g. expressed fusion) that could be used for MRD detection and monitoring; (3) Prognostic risk stratification: anomalies that change the patient's risk classification; (4) Diagnostic: molecular information that can change initial diagnosis and cancer predisposition variants.

TRICEPS multidisciplinary molecular tumor board (MMTB), included experts in pediatric oncology, genomics, bioinformatics, genetics, surgery and pathology. Based on the identified putative actionable alterations, the board reviewed the scientific literature, including results of clinical trials, case reports and biological data. A report outlining the actionable alterations found in each patient's cancer was discussed with the referring physician.

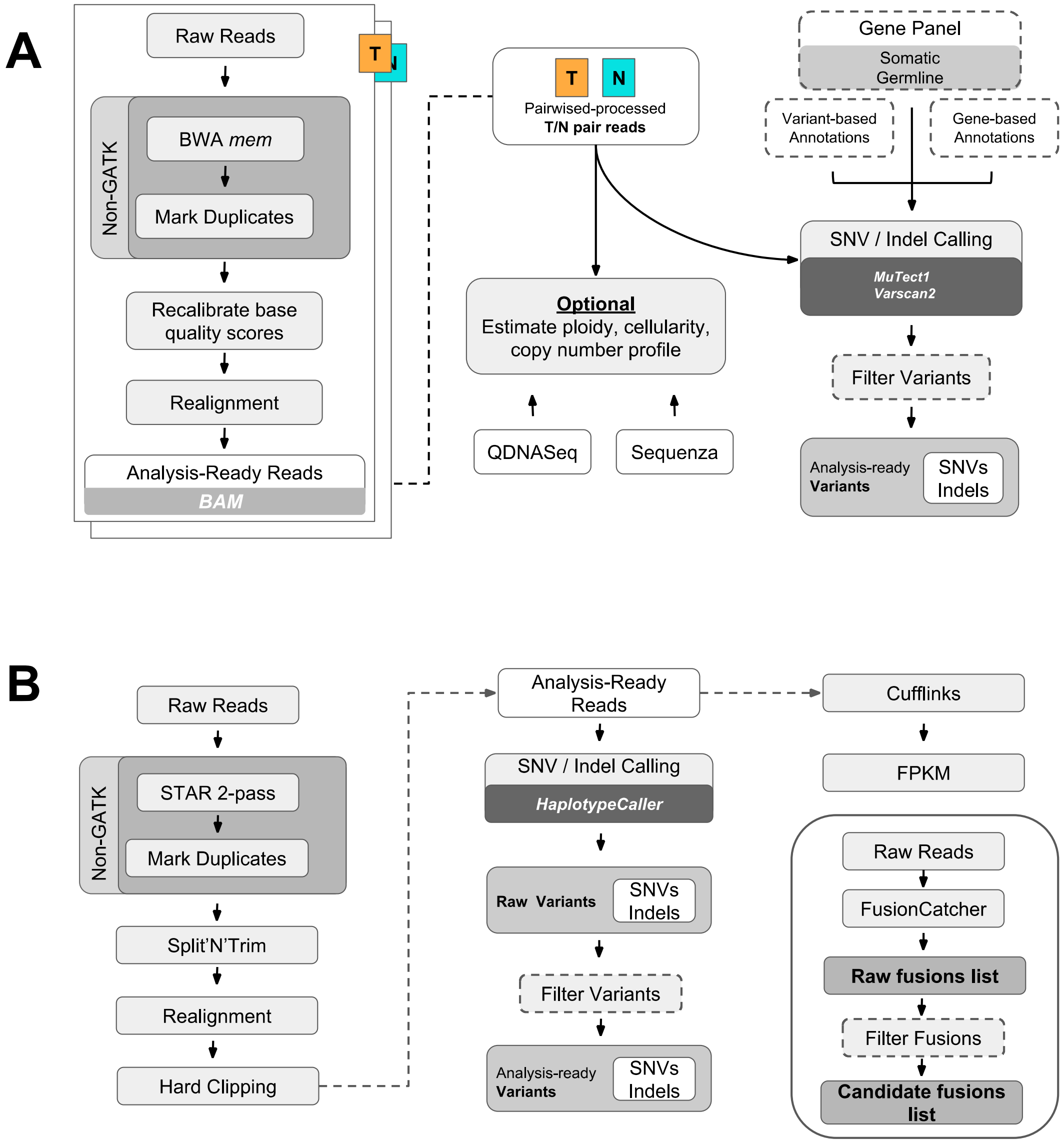

eFigure 1. Schematic Illustration of the Bioinformatics Pipelines Used for Genomic-based Molecular Profiling
(A) (B) Boxes represent the analyzing/cleaning steps for whole exome sequencing (WES) and RNA sequencing respectively. Details are given in the Materials and Methods section.

©2019 Khater F et al. *JAMA Network Open.*

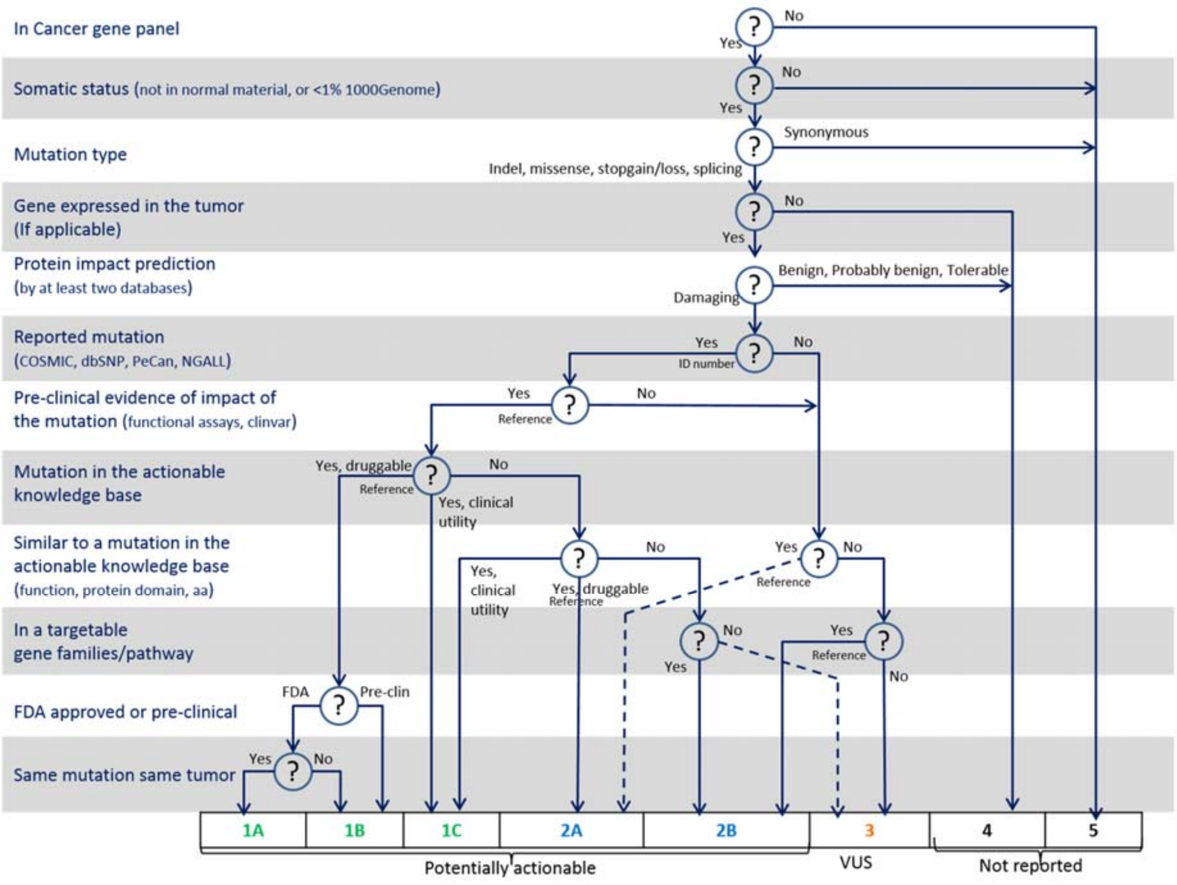

eFigure 2. Ranking of TRICEPS Actionable Alterations

Putative actionable alterations and relevant genomic findings were reported and ranked according to the illustration. 1A :Reported alteration, Clinical evidence, same disease, 1B : Reported alteration, Clinical/pre-clinical evidence, same/different disease, 1C : Reported alteration, Clinical/pre-clinical evidence, same/different disease, 2A : Reported/New alteration with similar function of a druggable one, same/different disease, 2B :Reported/New alteration in targetable gene family/pathway, same/different disease, 3 : Reported/New alteration, predicted damaging impact on protein, no Pre-clinical evidence of impact, 4 : In cancer related genes but without damaging impact nor expression in tumor, 5 : Not in cancer related genes or synonymous or frequent.

©2019 Khater F et al. *JAMA Network Open.*

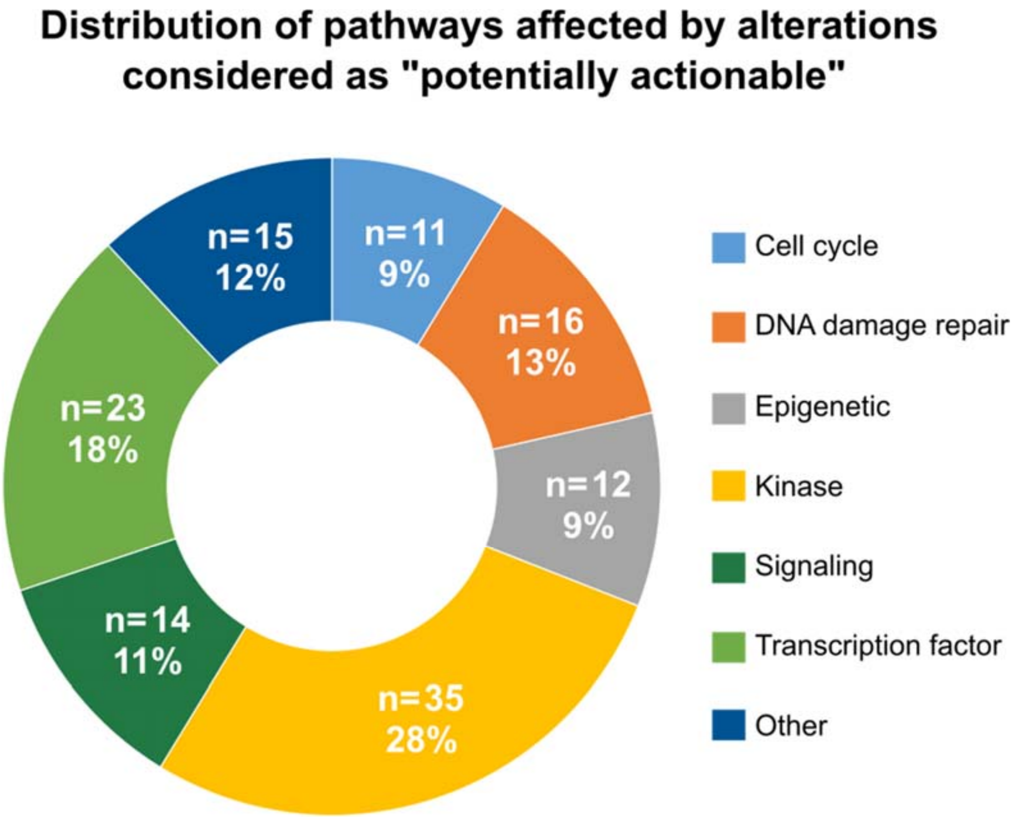

eFigure 3. Distribution of Molecular Alterations Considered as “Potentially Actionable” in Targeted Pathways

©2019 Khater F et al. *JAMA Network Open.*

eTable 1. Virtual 979 Cancer Gene Panel Used in TRICEPS to Detect Somatic Mutations
panel built from a compilation of genes present in the Catalogue Of Somatic Mutations In Cancer (COSMIC)[19], FoundationOne and FoundationOneHeme (http://foundationone.com) and MyCancer genome (http://www.mycancergenome.org).

ABI1	CD38	ERCC3	HDAC2	LOXL2	NTRK3	RALGDS	TBL1XR1
ABL1	CD3D	ERCC4	HDAC3	LPL	NUMA1	RANBP17	TBX3
ABL2	CD52	ERCC5	HDAC4	LPP	NUP214	RANBP2	TCEA1
ACKR3	CD58	ERG	HDAC5	LRIG3	NUP93	RAP1GDS1	TCF12
ACSL3	CD70	ERRFI1	HDAC6	LRP1B	NUP98	RARA	TCF3
ACSL6	CD74	ESR1	HDAC7	LRRK2	NUTM1	RASGEF1A	TCF4
ACTB	CD79A	ETNK1	HDAC8	LSM14A	NUTM2A	RB1	TCF7L2
ACVR1	CD79B	ETS1	HDAC9	LYL1	NUTM2B	RBM10	TCL1A
ACVR1B	CDC42EP1	ETV1	HERPUD1	LYN	OLIG2	RBM15	TCL6
ADORA3	CDC73	ETV4	HEY1	LZTR1	OMD	RBP2	TEC
AFF1	CDH1	ETV5	HGF	MAF	OPA1	RECQL4	TEK
AFF3	CDH11	ETV6	HIF1A	MAFB	P2RY8	REL	TEP1
AFF4	CDK1	EWSR1	HIP1	MAGED1	PAFAH1B2	RELN	TERC
AIM1	CDK12	EXOSC6	HIST1H1C	MAGI1	PAG1	RET	TERT
AKAP9	CDK2	EXT1	HIST1H1D	MAGI2	PAK3	RHOA	TET1
AKT1	CDK4	EXT2	HIST1H1E	MAL	PALB2	RHOH	TET2
AKT2	CDK5	EZH1	HIST1H2AC	MALAT1	PARK2	RICTOR	TFE3
AKT3	CDK6	EZH2	HIST1H2AG	MALT1	PARP1	RIT1	TFEB
ALDH2	CDK7	EZR	HIST1H2AL	MAML2	PASK	RMI2	TFG
ALK	CDK8	F2	HIST1H2AM	MAP2K1	PATZ1	RNASE1	TFPT
AMER1	CDK9	F5	HIST1H2BC	MAP2K2	PAX3	RNASE3	TFRC
ANGPT1	CDKN1A	FAF1	HIST1H2BJ	MAP2K4	PAX5	RNF2	TGFBR1
ANGPT2	CDKN1B	FAH	HIST1H2BK	MAP3K1	PAX7	RNF213	TGFBR2
APC	CDKN2A	FAM131B	HIST1H2BO	MAP3K13	PAX8	RNF43	THRAP3
APH1A	CDKN2B	FAM46C	HIST1H3B	MAP3K14	PBRM1	ROS1	TKT
AR	CDKN2C	FANCA	HIST1H4I	MAP3K6	PBX1	RPL10	TLL2
ARAF	CDS1	FANCC	HK3	MAP3K7	PC	RPL15	TLR5
ARFRP1	CDX2	FANCD2	HLA-A	MAPK1	PCBP1	RPL22	TLR8
ARHGAP26	CEACAM5	FANCE	HLF	MAX	PCLO	RPL5	TLX1
ARHGEF12	CEBPA	FANCF	HMGA1	MBD1	PCM1	RPN1	TLX3
ARID1A	CEBPZ	FANCG	HMGA2	MCL1	PCSK7	RPS6KB1	TMEM30A
ARID1B	CEP89	FANCL	HNF1A	MDM2	PDCD1	RPTOR	TMPRSS2
ARID2	CHCHD7	FAS	HNRNPA2B1	MDM4	PDCD11	RSPO2	TNFAIP3
ARNT	CHD2	FASN	HOOK3	MDS2	PDCD1LG2	RSPO3	TNFRSF10A
ASMTL	CHD4	FAT1	HOXA11	MECOM	PDE4DIP	RUNX1	TNFRSF10B

©2019 Khater F et al. *JAMA Network Open.*

ASNS	CHEK1	FAT4	HOXA13	MED12	PDGFB	RUNX1T1	TNFRSF11A
ASPSCR1	CHEK2	FBXO11	HOXA3	MEF2B	PDGFRA	RUNX2	TNFRSF14
ASXL1	CHIC2	FBXO30	HOXA9	MEF2C	PDGFRB	S1PR2	TNFRSF17
ATF1	CHN1	FBXO31	HOXC11	MEN1	PDK1	SBDS	TNFRSF8
ATG5	CIC	FBXW7	HOXC13	MET	PEMT	SCG2	TNFRSF9
ATIC	CIITA	FCGR2B	HOXD11	MIB1	PER1	SDC4	TNFSF11
ATL1	CKS1B	FCRL4	HOXD13	MITF	PFN1	SDHA	TNFSF13B
ATM	CLIP1	FEV	HRAS	MKI67	PGAP3	SDHAF2	TOP1
ATP1A1	CLP1	FGF1	HSD3B1	MKL1	PGR	SDHB	TOP2A
ATP2B3	CLTC	FGF10	HSP90AA1	MLF1	PHF1	SDHC	TOX4
ATR	CLTCL1	FGF14	HSP90AB1	MLH1	PHF2	SDHD	TP53
ATRX	CMC1	FGF19	HSP90B1	MLLT1	PHF6	SDS	TP63
AURKA	CNBP	FGF2	HSPB1	MLLT10	PHOX2B	SEC31A	TPM3
AURKB	CNOT3	FGF23	ICK	MLLT11	PICALM	SEMA4D	TPM4
AURKC	CNTRL	FGF3	ID3	MLLT3	PIGF	SETP2	TPMT
AXIN1	COL1A1	FGF4	IDH1	MLLT4	PIK3C2A	SEPT5	TPR
AXIN2	COL2A1	FGF6	IDH2	MLLT6	PIK3C2B	SEPT6	TRAF2
AXL	COX6C	FGFR1	IDO1	MMAB	PIK3C2G	SEPT9	TRAF3
B2M	CPS1	FGFR1OP	IGF1	MMACHC	PIK3CA	SERP2	TRAF5
B4GALNT1	CREB1	FGFR2	IGF1R	MMP9	PIK3CB	SET	TRAF7
BACH1	CREB3L1	FGFR3	IGF2	MN1	PIK3CD	SETBP1	TRAP1
BAP1	CREB3L2	FGFR4	IGF2R	MNX1	PIK3CG	SETD2	TRIM24
BARD1	CREBBP	FH	IKBKB	MPL	PIK3R1	SF3B1	TRIM27
BCL10	CRKL	FHIT	IKBKE	MRE11A	PIK3R2	SF3B2	TRIM33
BCL11A	CRLF2	FIP1L1	IKZF1	MRPL36	PIK3R3	SFPQ	TRIP11
BCL11B	CRTC1	FLCN	IKZF2	MS4A1	PIK3R4	SGK1	TRRAP
BCL2	CRTC3	FLG	IKZF3	MSH2	PIK3R5	SH2B3	TSC1
BCL2L1	CSF1	FLI1	IL2	MSH3	PIK3R6	SH2D1A	TSC2
BCL2L2	CSF1R	FLII	IL21R	MSH6	PIM1	SH3GL1	TSHR
BCL3	CSF3R	FLT1	IL2RA	MSI2	PLAG1	SLC1A2	TTF1
BCL6	CTCF	FLT3	IL3	MSLN	PLCG1	SLC34A2	TTL
BCL7A	CTLA4	FLT4	IL6ST	MSN	PLCG2	SLC45A3	TUSC3
BCL9	CTNNA1	FLYWCH1	IL7R	MST1R	PLK1	SLIT2	TYK2
BCOR	CTNNB1	FN1	INHBA	MTCP1	PML	SLTM	U2AF1
BCORL1	CUL3	FNBP1	INPP4B	MTOR	PMS1	SMAD2	U2AF2
BCR	CUX1	FNTA	INPP5D	MUC1	PMS2	SMAD3	UBA3
BIRC2	CXCR4	FNTB	IRF1	MUC16	POLD1	SMAD4	UBR5
BIRC3	CYLD	FOLH1	IRF2	MUM1	POLE	SMARCA1	UGT1A1
BIRC5	DAXX	FOLR1	IRF4	MUTYH	PORCN	SMARCA4	USP6
BIRC7	DCTN1	FOLR2	IRF8	MYB	POT1	SMARCB1	USP8
BLM	DDB2	FOLR3	IRS2	MYC	POU2AF1	SMARCD1	VEGFA
BMPR1A	DDIT3	FOXA1	ITK	MYCL	POU5F1	SMARCE1	VEGFB
BRAF	DDR2	FOXL2	JAK1	MYCN	PPARG	SMC1A	VEGFC
BRCA1	DDX10	FOXO1	JAK2	MYD88	PPFIBP1	SMC3	VHL
BRCA2	DDX3X	FOXO3	JAK3	MYH11	PPP1CB	SMO	VTI1A

©2019 Khater F et al. *JAMA Network Open.*

BRD2	DDX5	FOXO4	JARID2	MYH9	PPP2R1A	SMOX	WAS
BRD3	DDX6	FOXP1	JAZF1	MYO18A	PPP6C	SNCAIP	WDR90
BRD4	DEK	FRS2	JUN	MYO5A	PRCC	SND1	WEE1
BRIP1	DICER1	FSTL3	KAT6A	MYOD1	PRDM1	SNX29	WHSC1
BRSK1	DLL4	FUBP1	KAT6B	NAB2	PRDM16	SOCS1	WHSC1L1
BTG1	DNAJB1	FUS	KCNJ5	NACA	PREX2	SOCS2	WIF1
BTG2	DNM2	FZD7	KDM1A	NAIP	PRF1	SOCS3	WISP3
BTK	DNMT3A	FZD8	KDM2B	NAPB	PRG4	SOX10	WRN
BTLA	DOT1L	FZR1	KDM4C	NBN	PRKACA	SOX2	WT1
BUB1B	DPYD	G6PD	KDM5A	NCKIPSD	PRKAR1A	SOX9	WWTR1
C11orf30	DRG1	GABRA6	KDM5C	NCOA1	PRKCA	SPECC1	XBP1
C15orf65	DTX1	GADD45B	KDM6A	NCOA2	PRKCB	SPEN	XIAP
C2orf44	DUSP2	GAS7	KDR	NCOA4	PRKCG	SPOP	XPA
CACNA1D	DUSP22	GATA1	KDSR	NCOR1	PRKCI	SPTA1	XPC
CAD	DUSP9	GATA2	KEAP1	NCOR2	PRKDC	SRC	XPO1
CALR	DUX4	GATA3	KEL	NCSTN	PRRX1	SRGAP2	YPEL5
CAMTA1	EBF1	GATA4	KIAA1549	NDRG1	PRSS8	SRGAP3	YWHAE
CANT1	ECT2L	GATA6	KIAA1598	NEDD8	PSIP1	SRSF2	YY1AP1
CAP1	EED	GDNF	KIF11	NF1	PSMD2	SRSF3	ZBTB16
CARD11	EGFL7	GID4	KIF5B	NF2	PTCH1	SS18	ZBTB2
CARS	EGFR	GLI1	KIT	NFATC2	PTEN	SS18L1	ZCCHC8
CASC5	EIF3E	GMPS	KLF4	NFE2L2	PTK2	SSX1	ZFHX3
CASP8	EIF4A2	GNA11	KLF6	NFIB	PTK6	SSX2	ZMYM2
CBFA2T3	ELF4	GNA12	KLHL6	NFKB2	PTK7	SSX4	ZMYM3
CBFB	ELK4	GNA13	KLK2	NFKBIA	PTPN11	STAG2	ZNF217
CBL	ELL	GNAQ	KMT2A	NFKBIE	PTPN13	STAT3	ZNF24
CBLB	ELN	GNAS	KMT2B	NIN	PTPN2	STAT4	ZNF331
CBLC	ELP2	GOLGA5	KMT2C	NKX2-1	PTPN6	STAT5A	ZNF384
CCDC6	EML4	GOPC	KMT2D	NLRP2	PTPRB	STAT5B	ZNF521
CCNB1IP1	ENG	GPC3	KRAS	NOD1	PTPRC	STAT6	ZNF703
CCND1	EP300	GPHN	KTN1	NONO	PTPRK	STEAP1	ZRSR2
CCND2	EPAS1	GPNMB	LAP3	NOTCH1	PTPRO	STIL	
CCND3	EPHA3	GPR124	LASP1	NOTCH2	PVRL4	STK11	
CCNE1	EPHA5	GRIN2A	LCK	NOTCH3	PWWP2A	STRN	
CCR4	EPHA7	GRIP1	LCP1	NOV	QKI	SUFU	
CCT6B	EPHB1	GRM3	LEF1	NPM1	RABEP1	SUZ12	
CD19	EPHB4	GSK3B	LHFP	NR4A3	RAC1	SYK	
CD22	EPOR	GTSE1	LIFR	NRAS	RAD1	TACSTD2	
CD248	EPS15	GUCY2C	LIG3	NRG1	RAD21	TAF1	
CD27	ERBB2	H3F3A	LINC0059<br>8	NRP1	RAD50	TAF15	
CD274	ERBB3	H3F3B	LMNA	NSD1	RAD51	TAL1	
CD33	ERBB4	HDAC1	LMO1	NT5C2	RAD51B	TAL2	
CD36	ERC1	HDAC10	LMO2	NTRK1	RAD54L	TAZ	
CD37	ERCC2	HDAC11	LONP1	NTRK2	RAF1	TBK1	

©2019 Khater F et al. *JAMA Network Open.*

eTable 2. Virtual 112 Cancer Predisposition Gene Panel Used in TRICEPS to Detect Germline Variants [18]

ALK	*DKC1*	*HNF1A*	*PTEN*	*SLX4*
APC	*EPCAM*	*HRAS*	*RAD51C*	*SMAD4*
ATM	*ERCC2*	*KIT*	*RAD51D*	*SMARCA4*
BAP1	*ERCC3*	*LZTR1*	*RB1*	*SMARCB1*
BLM	*ERCC4*	*MAX*	*RECQL4*	*SMARCE1*
BMPR1A	*ERCC5*	*MEN1*	*RET*	*STK11*
BRCA1	*EXT1*	*MET*	*RHBDF2*	*SUFU*
BRCA2	*EXT2*	*MLH1*	*RPL5*	*TERC*
BRIP1	*EZH2*	*MSH2*	*RPL11*	*TERT*
BUB1B	*FANCA*	*MSH6*	*RPL26*	*TINF2*
CDH1	*FANCB*	*MUTYH*	*RPL35A*	*TMEM127*
CDK4	*FANCC*	*NBN*	*RPS7*	*TP53*
CDKN1B	*FANCD2*	*NF1*	*RPS10*	*TSC1*
CDKN1C	*FANCE*	*NF2*	*RPS17*	*TSC2*
CDKN2A	*FANCF*	*NHP2*	*RPS19*	*VHL*
CEBPA	*FANCG*	*NOP10*	*RPS24*	*WAS*
CEP57	*FANCI*	*PALB2*	*RPS26*	*WRN*
CHEK2	*FANCL*	*PDGFRA*	*RUNX1*	*WT1*
CYLD	*FANCM*	*PHOX2B*	*SBDS*	*XPA*
DDB2	*FH*	*PMS2*	*SDHAF2*	*XPC*
DICER1	*FLCN*	*PRKAR1A*	*SDHB*	
DIS3L2	*GATA2*	*PTCH1*	*SDHC*	
EGFR	*GPC3*	*PTCH2*	*SDHD*	

eTable 3. Summary of the Genomic Sequencing Initiatives in Pediatric Oncology

Study Name	Peds-MiOncoSeq	ICAT	BASIC3	INFORM	MBB Program	PIPseq	MOSCATO-01	NCI Study	TRICEPS
**Lead Institution**	University of Michigan	Dana-Farber Cancer Institute	Baylor College of Medicine	German cancer research center	Institut Curie, Paris	Columbia University, Medical Center	Gustave-Roussy	Pediatric Oncology Branch	CHU Sainte-Justine
**Tumor types**	Solid & Brain tumors; Hematol malignancies	Solid tumors	Solid & Brain tumors	Solid & Brain tumors; Hematol malignancies	Solid & Brain tumors	Solid & Brain tumors; Hematol malignancies	Solid & Brain tumors	Solid tumors	Solid & Brain tumors; Hematol malignancies
**Targeted population**	Relapse/ refractory; Rare cancer	Relapse/ refractory; High-risk new diagnosis	New Diagnosis	Relapse/ refractory	Relapse/ refractory ; High-risk new diagnosis	Relapse/ refractory; High-risk new diagnosis	Relapse/ refractory	Relapse/ refractory	Relapse/ refractory
**Patients enrolled**	102	101	150	57	60	107	75	64	84
**Patients analyzed**	91	89	121	52	58	101	69	59	62
**Age Range at diagnostic**	<22 yrs (? ≤18)	<30 yrs (75 ≤18)	≤17 yrs	<40 yrs (40 ≤18)	<22 yrs (49 ≤18)	≤18	<25 yrs (62 ≤18)	<25 yrs (51 ≤ 18)	<22 yrs (80≤18)
**Molecular Profiling**	WES (150X); RNA-Seq PolyA (67M reads)	Oncomap panel (41 genes); NGS Oncopanel (275 genes); aCGH	WES (272X)	WES (165X); WGS (3.4X); RNA-seq PolyA/Total (220M reads)	NGS Panel (50 genes); aCGH	WES (150X); RNA-Seq total (50M reads)	WES (125X); RNA-seq polyA (117M reads); aCGH	WES (75X); RNA-seq polyA/total (227M reads); SNP array	WES (250X); RNA-seq total (150M reads)
**Data reported**	Somatic & germline SNVs; CNAs; gene fusions	Somatic SNVs; CNAs	Somatic & germline SNVs	Somatic & germline SNVs; CNAs; gene fusions	Somatic SNVs; CNAs	Somatic & germline SNVs; CNAs; gene fusions	Somatic & germline SNVs; CNAs; gene fusions	Somatic & germline SNVs; CNAs; gene fusions	Somatic & germline SNVs; CNAs; gene fusions
**Actionable alterations (%)**	46	43	39	50	40	66	61	51	87
**Reference**	20	21	18	22	23	24	25	26	Khater et al. (this study)

Figure and Table Legends

eFigure 1: Schematic illustration of the bioinformatics pipelines used for genomic-based molecular profiling. (A) (B) Boxes represent the analyzing/cleaning steps for whole exome sequencing (WES) and RNA sequencing respectively. Details are given in the Materials and Methods section

eFigure 2 : Ranking of TRICEPS actionable alterations. Putative actionable alterations and relevant genomic findings were reported and ranked according to the illustration. 1A :Reported alteration, Clinical evidence, same disease, 1B : Reported alteration, Clinical/pre-clinical evidence, same/different disease, 1C : Reported alteration, Clinical/pre-clinical evidence, same/different disease, 2A : Reported/New alteration with similar function of a druggable one, same/different disease, 2B :Reported/New alteration in targetable gene family/pathway, same/different disease, 3 : Reported/New alteration, predicted damaging impact on protein, no

©2019 Khater F et al. *JAMA Network Open.*

Pre-clinical evidence of impact, 4 : In cancer related genes but without damaging impact nor expression in tumor, 5 : Not in cancer related genes or synonymous or frequent.

eFigure 3: Distribution of molecular alterations considered as "potentially actionable" in targeted pathways.

eTable 1: Virtual 979 Cancer gene panel used in TRICEPS to detect somatic mutations. Panel built from a compilation of genes present in the Catalogue Of Somatic Mutations In Cancer (COSMIC)[19], FoundationOne and FoundationOneHeme (http://foundationone.com) and MyCancer genome (http://www.mycancergenome.org).

eTable 2: Virtual 112 cancer predisposition gene panel used in TRICEPS to detect germline variants [18]

eTable 3: Genomic sequencing initiatives in pediatric oncology. adapted from

References

1. Spinella JF, Cassart P, Richer C, et al. Genomic characterization of pediatric T-cell acute lymphoblastic leukemia reveals novel recurrent driver mutations. *Oncotarget.* 2016;7(40):65485-65503.
2. Li H, Durbin R. Fast and accurate short read alignment with Burrows-Wheeler transform. *Bioinformatics (Oxford, England).* 2009;25(14):1754-1760.
3. McKenna A, Hanna M, Banks E, et al. The Genome Analysis Toolkit: a MapReduce framework for analyzing next-generation DNA sequencing data. *Genome Res.* 2010;20(9):1297-1303.
4. Koboldt DC, Chen K, Wylie T, et al. VarScan: variant detection in massively parallel sequencing of individual and pooled samples. *Bioinformatics (Oxford, England).* 2009;25(17):2283-2285.
5. Scheinin I, Sie D, Bengtsson H, et al. DNA copy number analysis of fresh and formalin-fixed specimens by shallow whole-genome sequencing with identification and exclusion of problematic regions in the genome assembly. *Genome Res.* 2014;24(12):2022-2032.
6. Dobin A, Davis CA, Schlesinger F, et al. STAR: ultrafast universal RNA-seq aligner. *Bioinformatics (Oxford, England).* 2013;29(1):15-21.
7. Nicorici D, Satalan M, Edgren H, et al. FusionCatcher-a tool for finding somatic fusion genes in paired-end RNA-sequencing data. *bioRxiv.* 2014:011650.
8. Kent WJ, Sugnet CW, Furey TS, et al. The human genome browser at UCSC. *Genome research.* 2002;12(6):996-1006.
9. Kent WJ. BLAT—the BLAST-like alignment tool. *Genome research.* 2002;12(4):656-664.

©2019 Khater F et al. *JAMA Network Open.*

10. Genomes Project C, Auton A, Brooks LD, et al. A global reference for human genetic variation. *Nature.* 2015;526(7571):68-74.
11. Wang K, Li M, Hakonarson H. ANNOVAR: functional annotation of genetic variants from high-throughput sequencing data. *Nucleic Acids Res.* 2010;38(16):e164.
12. Ramos AH, Lichtenstein L, Gupta M, et al. Oncotator: cancer variant annotation tool. *Hum Mutat.* 2015;36(4):E2423-2429.
13. Ng PC, Henikoff S. SIFT: Predicting amino acid changes that affect protein function. *Nucleic Acids Res.* 2003;31(13):3812-3814.
14. Adzhubei I, Jordan DM, Sunyaev SR. Predicting functional effect of human missense mutations using PolyPhen-2. *Curr Protoc Hum Genet.* 2013;Chapter 7:Unit7 20.
15. Kircher M, Witten DM, Jain P, O'Roak BJ, Cooper GM, Shendure J. A general framework for estimating the relative pathogenicity of human genetic variants. *Nat Genet.* 2014;46(3):310-315.
16. Spinella JF, Mehanna P, Vidal R, et al. SNooPer: a machine learning-based method for somatic variant identification from low-pass next-generation sequencing. *BMC Genomics.* 2016;17(1):912.
17. Favero F, Joshi T, Marquard AM, et al. Sequenza: allele-specific copy number and mutation profiles from tumor sequencing data. *Ann Oncol.* 2015;26(1):64-70.
18. Parsons DW, Roy A, Yang Y, et al. Diagnostic Yield of Clinical Tumor and Germline Whole-Exome Sequencing for Children With Solid Tumors. *JAMA Oncol.* 2016.
19. Bamford S, Dawson E, Forbes S, et al. The COSMIC (Catalogue of Somatic Mutations in Cancer) database and website. *Br J Cancer.* 2004;91(2):355-358.
20. Mody RJ, Wu YM, Lonigro RJ, et al. Integrative Clinical Sequencing in the Management of Refractory or Relapsed Cancer in Youth. *JAMA.* 2015;314(9):913-925.
21. Harris M, Bhatti Y, Darzi A. Does the Country of Origin Matter in Health Care Innovation Diffusion? *JAMA.* 2016;315(11):1103-1104.
22. Worst BC, van Tilburg CM, Balasubramanian GP, et al. Next-generation personalised medicine for high-risk paediatric cancer patients - The INFORM pilot study. *Eur J Cancer.* 2016;65:91-101.
23. Pincez T, Clement N, Lapouble E, et al. Feasibility and clinical integration of molecular profiling for target identification in pediatric solid tumors. *Pediatr Blood Cancer.* 2017;64(6).
24. Oberg JA, Glade Bender JL, Sulis ML, et al. Implementation of next generation sequencing into pediatric hematology-oncology practice: moving beyond actionable alterations. *Genome Med.* 2016;8(1):133.
25. Harttrampf AC, Lacroix L, Deloger M, et al. Molecular Screening for Cancer Treatment Optimization (MOSCATO-01) in Pediatric Patients: A Single-Institutional Prospective Molecular Stratification Trial. *Clin Cancer Res.* 2017;23(20):6101-6112.
26. Chang W, Brohl AS, Patidar R, et al. MultiDimensional ClinOmics for Precision Therapy of Children and Adolescent Young Adults with Relapsed and Refractory Cancer: A Report from the Center for Cancer Research. *Clin Cancer Res.* 2016;22(15):3810-3820.

©2019 Khater F et al. *JAMA Network Open.*
